# Investigating Irrational Beliefs, Cognitive Appraisals, Challenge and Threat, and Affective States in Golfers Approaching Competitive Situations

**DOI:** 10.3389/fpsyg.2019.02295

**Published:** 2019-10-10

**Authors:** Nanaki J. Chadha, Martin J. Turner, Matthew J. Slater

**Affiliations:** Life Sciences and Education, Staffordshire University, Stoke-on-Trent, United Kingdom

**Keywords:** stress, REBT, performance, pressure, imagined, CBT

## Abstract

On approach to competitive situations, affective states (emotions and anxiety) occur through the complex interaction of cognitive antecedents. Researchers have intimated that irrational beliefs might play an important role in the relationship between cognitive appraisals and affective states, but has ignored challenge and threat. In the current research, we examine the interaction between cognitive appraisals, irrational beliefs, and challenge and threat to predict golfers’ pre-competitive affective states. We adopted a cross-sectional atemporal design to examine how golfers approached two different competitive situations: imagined imminent golf competition (phase 1), and actual future golf competition (phase 2). Path analysis revealed how cognitive appraisals, irrational beliefs, and challenge and threat interact to predict affective states among golfers. Serial atemporal multiple mediation analysis indicated that the relationships between cognitive appraisals and affective states were mediated by irrational beliefs and challenge and threat. Further, some differences were revealed between phase 1 and phase 2 in the serial multiple atemporal mediation results with regard to challenge. That is, at phase 1 no significant serial mediation was found for any affective outcomes, but at phase 2 significant serial mediation was found for all affective states, showing that irrational beliefs and challenge serial mediated the associations between cognitive appraisals and affective states. The finding that mediation and bivariate associations differed across phase 1 and phase 2 is echoed in the phase 1-phase 2 tests of differences. The current research makes a theoretical advancement by elucidating in more detail the complex interaction between cognitive antecedents and mediators of affective states. Specifically, the inclusion of challenge and threat alongside irrational beliefs and cognitive appraisals is an important theoretical advancement that builds on work inside of sport literature (e.g., Dixon et al., 2016) and outside of sport literature (e.g., David et al., 2002, 2005), as this constellation of theoretically related antecedents of affective states has not been examined together in the extant research.

## Introduction

For individuals taking part in sport, the anticipation time prior to stressful situations such as a sporting competition (Neil et al., 2011) is often daunting due to an over emphasis on winning and uncertainty of the outcome (Folkman and Lazarus, 1985). Athletes’ pre-competitive anticipatory psychological states have been the focus of much research, and competition anxiety is one of the most studied areas in the discipline of sport psychology (Mellalieu et al., 2006). There are a number of frameworks that attempt to explain the occurrence of pre-competitive emotions (Jones and Uphill, 2012), but one underexplored framework that is growing in the sport literature (Turner, 2016) is Rational Emotive Behavioral Therapy (REBT; Ellis, 1957).

REBT is considered to be the original cognitive behavioral therapy (CBT), and was developed by Albert Ellis in 1955, inspired by ancient philosophers, particularly the Stoic philosopher Epictetus (1948) who proclaimed in *The Enchiridion*: “men are not disturbed by things, but by the view which they take of them.” Ellis (1994) developed a framework for understanding and treating psychological disturbance known as the GABC framework. In this framework, individual goals, values, and desires (G), that are thwarted or obstructed by events and situations (A), can trigger healthy or unhealthy emotional and behavioral consequences (C), depending on one’s beliefs (B) about the self, others, and the world in relation to the situation (A). If an individual’s beliefs are rational (flexible, logical, and non-extreme) then healthy emotions and adaptive behaviors will occur. In contrast, if an individual’s beliefs are irrational (rigid, illogical, and extreme) then unhealthy emotions and maladaptive behaviors will occur (Szentagotai and Jones, 2010). As such, irrational beliefs have attracted much research attention (e.g., Visla et al., 2016).

Within REBT, irrational and rational beliefs are the core constructs that mediate between what we experience, and our emotional responses. Since its inception in 1955 (Ellis, 1957), REBT has included irrational beliefs as the fundamental cognitions that determine psychological ill-being. In sport and exercise literature, irrational beliefs as posited in REBT have been the subject of enquiry more recently (Turner et al., 2019a), and data indicates that irrational beliefs are a risk factor for mental illness in athletes (Turner, 2016). In the current paper, we seek to gain a deeper and more complex understanding of how irrational beliefs determine athlete affective states (emotions and anxiety).

In REBT, it is suggested that individuals often adopt irrational beliefs in situations that are of utmost importance to them. Irrational beliefs have been consistently associated with various types of emotional distress (Visla et al., 2016), with the positive relationship between irrational beliefs and anxiety being particularly strong (*r* = 0.41). Importantly, the association between irrational beliefs and anxiety is stronger when a stressful event is real, actually present, and is personally relevant, as opposed to being experimentally induced, absent, and not personally relevant. In sport, higher irrational beliefs have been found to be related to greater emotional and physical exhaustion (Turner and Moore, 2015), and anxiety, anger, and depression (Turner et al., 2019b). Also, irrational beliefs have been targeted for intervention in athletes experiencing heightened anxiety (Turner and Barker, 2013; Turner et al., 2018a).

Although in the extant literature irrational beliefs have been found to be associated with dysfunctional emotions and maladaptive behaviors (see Turner, 2016 for a review), the precise mechanisms that explain how irrational beliefs lead to emotional and behavioral dysfunction has not yet been fully elucidated. Over the years REBT has grown into a well-established CBT, but it remains less visible in the mainstream study of emotion due to lack of experimental rigor (Still, 2001; David et al., 2002; Padesky and Beck, 2003). There is a growing body of research that places irrational beliefs within the conceptual framework of cognitive appraisal theory (CAT; Lazarus, 1991; David et al., 2002, 2005) in order to advance Ellis’s cognitive theory of emotion. Therefore, the main purpose of the current study is to examine irrational beliefs as part of cognitive appraisals in the prediction of pre-competitive affective states.

Past literature has intimated that irrational beliefs might play an important role in cognitive appraisals (David et al., 2002, 2005). According to Lazarus’ CAT (Lazarus and Folkman, 1984; Lazarus, 1991; Smith and Lazarus, 1993), information processing includes a transaction between the goals of the individual and the representation of environmental encounters. This transaction can be appraised as harmful, beneficial, threatening or challenging. The CAT comprises primary appraisals, which are concerned with the extent to which the encounter is relevant to one’s well-being, and secondary appraisals which concerns one’s resources and options for coping with the encounter (Smith and Lazarus, 1993). Specifically, primary appraisal includes motivational relevance (MR; evaluation of the extent to which the encounter is relevant to one’s goals) and motivational congruence (MC; evaluation of the extent to which the encounter is consistent with one’s goals). In anticipation of stressors, the components of secondary appraisal are problem-focused coping potential (PFC; evaluations of one’s ability to act directly on the situation to bring it in accord with one’s goals), and emotion focused coping potential (EFC; evaluations of one’s ability to psychologically adjust to the situation by altering one’s interpretations, desires, or beliefs; Smith and Lazarus, 1993). The primary and secondary appraisals combine to form different core-relational themes that result in emotions. For anxiety, the core relational theme is uncertain, existential threat (Lazarus, 1991) where primary appraisals of high MR and low MC combine with secondary appraisals of low EFC (Smith and Lazarus, 1993).

Researchers have explored the links between irrational beliefs and cognitive appraisals, finding that anxiety is most effectively predicted by a combination of high MR, low MC, low EFC, and irrational beliefs (David et al., 2002, 2005). Clearly, there are some demonstrable relationships between the concepts of irrational beliefs proposed by Ellis, and CAT proposed by Lazarus. Ellis and Lazarus recognized this potential relationship in their works, with Ellis recognizing the influence of Lazarus on his thinking (Ellis, 1994), and with Lazarus explicitly addressing the overlap between REBT and the Lazarusian CAT (Lazarus, 1989). To explain the potential links between REBT and the CAT, Ziegler (2001) suggests that cognitive appraisals (both primary and secondary) are thoroughly couched in, and interconnected with, beliefs in the GABC model. For example, a golfer is anticipating the tee-off for an important competition with a lucrative reward (reflecting G in the REBT model, and MR in the CAT). The golfer has not competed in such a prestigious event before and is unsure whether he will perform well (reflecting A in the REBT model, and low MC in the CAT) and believes that he absolutely must perform well and he could not tolerate underperforming (reflecting irrational beliefs in the REBT model). Because the prospect of underperforming (A) is rendered highly dangerous to his goals (G) by the irrational beliefs, the golfer is likely to appraise the situation as a threat (Lazarus, 1999). If the golfer believes that he cannot psychologically adjust to the encounter (low EFC), and is not flexible in his coping abilities (Ziegler, 2001), then he is more likely to experience dysfunctional anxiety (David et al., 2002) in anticipation of the tee-off. Importantly, cognitive appraisals and irrational beliefs are seen as co-occurring simultaneously rather than occurring in a sequential and fixed order.

Within a sporting context, researchers have investigated the association between irrational beliefs and challenge and threat, finding irrational beliefs to be positively associated with threat and no association to be found with challenge (Dixon et al., 2016). Similarly, another study (Evans et al., 2018) found that soccer athletes who received a rational team talk (promoting rational beliefs) at half-time reported significantly lower threat compared to athletes who received an irrational team talk (promoting irrational beliefs). Research has also examined the effect of irrational and rational beliefs on performance within golf (Turner et al., 2018a, b). One study (Turner et al., 2018b) found that when golfers used rational self-talk they performed more accurately in a putting task than when they used irrational self-talk. Similarly, Turner et al. (2018a) used an REBT intervention with amateur golfers and found that as irrational beliefs decreased so to did golf-specific anxiety and in addition, golf performance improved. However, this fledgling research fails to examine how irrational beliefs and challenge and threat interact to predict competitive affective states. In the present study, cognitive appraisals, irrational beliefs, and challenge and threat are assessed in relation to upcoming competitive situations. Based on past research, it is the combination of these psychological constructs that gives rise to emotions in competitive situations (Neil et al., 2011).

The constructs of challenge and threat have been the subject of growing research in sport literature (e.g., Blascovich et al., 2004), spawning theories of challenge and threat that attempt to predict athletic performance (Jones et al., 2009; Vine et al., 2016). Challenge and threat are important constructs in Lazarus’s appraisal process and are labeled as relational meanings in his appraisal theory (Lazarus, 2000). Threat appraisal refers to evaluation of future harm or loss; whereas challenge appraisal occurs when an individual perceives a future gain (Lazarus, 1991). In extant theory, challenge and threat result in emotional responses, where challenge is said to be associated with more positive emotions, whereas threat is associated with more negative emotions (Skinner and Brewer, 2004; Jones et al., 2009). Furthermore, positive emotions are proposed to be interpreted as facilitative for performance in challenge whereas negative emotions as debilitative in threat (Skinner and Brewer, 2004; Jones et al., 2009). With regards to anxiety, research evidence demonstrates that threat is positively associated with greater cognitive and somatic anxiety and a more debilitative interpretation of anxiety compared to challenge (Williams et al., 2010; Quested et al., 2011; Moore et al., 2012). Therefore, challenge and threat are important antecedents to affective states that should be studied alongside cognitive appraisals, and irrational beliefs.

The current research is the first to investigate and understand how affective states occur through the complex interaction of antecedent cognitive appraisals, irrational beliefs and challenge and threat within a specific sporting population. This integrative examination might facilitate a more complete understanding of how affective states occur through the complex interaction of cognitive antecedents.

### The Current Research

**FIGURE 1:**
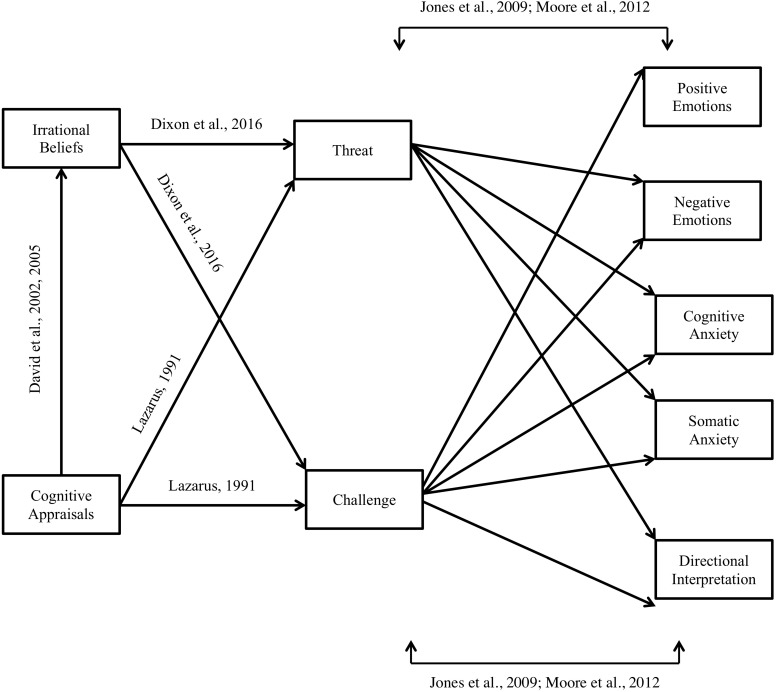
Proposed theoretical model.

The main aim of the current study is to examine the interaction between, cognitive appraisals, irrational beliefs, and challenge and threat, to predict pre-competitive affective states. To achieve this main aim, two study phases are reported; phase 1 meets the main aim in an imagined imminent golf competition, and phase 2 meets the main aim in an actual future golf competition. For the two phases, we illustrate our hypotheses in Figure 1, which are informed and supported by past research. Based on past research, it is hypothesized that (H1) golfers’ cognitive appraisals will be negatively associated with irrational beliefs (David et al., 2002, 2005), (H2) high irrational beliefs will be positively associated with threat and negatively with challenge (Dixon et al., 2016), (H3) cognitive appraisals will be negatively associated with threat and positively with challenge (Lazarus, 1999), (H4) challenge will be positively associated with positive emotions, and threat will be positively associated with negative emotions (Jones et al., 2009), (H5) threat will be positively associated with cognitive and somatic anxiety, and challenge will be negatively associated with cognitive and somatic anxiety (Moore et al., 2012), and (H6) threat will be negatively associated with facilitative perceptions of anxiety, and challenge will be positively associated with facilitative perceptions of anxiety (Quested et al., 2011). It is also hypothesized that (H7) the relationship between cognitive appraisals and affective states will be mediated by irrational beliefs (David et al., 2002, 2005) and challenge and threat (Jones et al., 2009). Further, on the basis of meta-analytical data (Visla et al., 2016) where stronger associations were found between irrational beliefs and affective states during a real-stressor, we hypothesize that (H8) the associations between target variables will be stronger in phase 2 than in phase 1. Lastly, we examine differences in variables between study phases 1 and 2, and hypothesize that (H9) in phase 2 golfers will report greater cognitive appraisals, irrational beliefs, threat and affective states and lower challenge, positive emotions and facilitative perceptions of anxiety in comparison to phase 1.

## Materials and Methods

### Participants

In phase 1, 287 participants (*Male* = 232, *Female* = 55; *M*age = 38.7 ± 15.20) with a golf handicap between 0 and 31 (*M*handicap = 8.85 ± 7.13) took part in the study. The participants encompassed Indians (*n* = 220), British (*n* = 41) and other ethnic origins (*n* = 26). They had an average of 11.85 years (± 8.31) golfing experience and were competing at a club (*n* = 115), amateur (*n* = 120) and professional (*n* = 52) level. In phase 2, 212 golfers (*Male* = 169, *Female* = 43; *M*age = 38.55 ± 15.08) with a handicap between 0 and 31 (*M*handicap = 8.68 ± 7.16) completed the study. The participants encompassed Indians (*n* = 161), British (*n* = 30) and other ethnic origins (*n* = 21). They had an average of 12.28 years (± 8.38) of golfing experience and were competing at the club (*n* = 83), amateur (*n* = 86) and professional (*n* = 43) level. No incentive was offered to the participants for taking part in the research. Ethical approval was granted from the ethics committee of Staffordshire University and individual informed consent was obtained prior to data collection. The participants were recruited by contacting local golf clubs on their willingness to participate in the research project. The lead author approached golf clubs and golf organizations in India to recruit golfers. Further, the distribution of an online survey resulted in snowball sampling that helped in the recruitment of golfers.

### Measures

#### Irrational Performance Beliefs

The irrational Performance Beliefs Inventory (iPBI; Turner et al., 2018) was used as a performance specific measure of irrational beliefs. It comprises 28-items representing four core irrational beliefs; primary belief and three secondary beliefs (Ellis and Dryden, 1997). The primary irrational belief is stated to be demandingness (DEM), which refers to rigid, absolutistic requirements expressed in the form of “musts,” “shoulds,” and “oughts” (e.g., “I must attain my goals”). The three secondary irrational beliefs comprise of awfulizing (AWF), low frustration tolerance (LFT) and depreciation (DEP). AWF refers to the beliefs that an individual holds where unpleasant situations are assessed in the greatest negative manner (e.g., “If I don’t attain my goals it is awful”). LFT reflects an individuals evaluation that they are absolutely incapable of enduring a given situation, accompanied with the view that they will not experience any happiness if what they want does not exist (e.g., “If I don’t attain my goals I can’t stand it”), and DEP appears when individuals tend to be excessively critical about themselves, others or the world when they fail to live up to their self-imposed demands (e.g., “If I don’t attain my goals, I am a complete failure”; Ellis, 1994). The responses are made on a 5-point Likert-scale ranging from 1 (*strongly disagree*) to 5 (*strongly agree*) to a series of performance belief statements. The iPBI has previously been used with athletes (Turner et al., 2019b) including golfers (Turner et al., 2018a) and has demonstrated good internal validity and reliability among sporting populations (Turner and Allen, 2018). However, due to a novel and relatively homogenous sample population in the current study, confirmatory factor analysis (CFA) was conducted to test the four-factor structure of the iPBI. One item from DEM showing factor loading less than 0.40 was eliminated from further analyses (Comrey and Lee, 1992; Tabachnick and Fidell, 2007). Cronbach’s alphas from the current sample were 0.76 for DEM, 0.84 for AWF, 0.87 for LFT, and 0.87 for DEP.

#### Cognitive Appraisals

The primary and secondary cognitive appraisals were assessed with five single-item questions used in previous research (David et al., 2002), modified from Smith and Lazarus (1993). The single-item questions were answered on a 11-points Likert-scale ranging from 1 (*not at all*) to 11 (*extremely*). The measure assesses motivational relevance (MR), motivational congruence (MC; 2-items), problem-focused coping potential (PFC), and emotion-focused coping (EFC). A total cognitive appraisal score was obtained by calculating the mean score of all the items. Higher cognitive appraisals indicated more positive appraisals.

#### Challenge and Threat

The Challenge and Threat in Sport scale (CAT-Sport; Rossato et al., 2016), comprises 12-items representing two subscales; challenge and threat. The responses are made on a 6-point Likert-scale ranging from 1 (*totally disagree*) to 6 (*totally agree*) in anticipation of a competition. The CAT-Sport has only recently been developed, so confirmatory factor analysis (CFA) was conducted to test the two-factor structure. One item from challenge displaying factor loading less than 0.40 was eliminated from further analyses (Comrey and Lee, 1992; Tabachnick and Fidell, 2007). The CAT-Sport has previously demonstrated good internal validity and reliability in athlete populations (Rossato et al., 2016) and the Cronbach’s alphas from the current sample were 0.90 for threat, and 0.77 for challenge in phase 1, and 0.91 for threat and 0.82 for challenge in phase 2.

#### Emotion

The Positive and Negative Affect Schedule (PANAS; Watson et al., 1988) incorporates two 10-item subscales based on a bi-dimensional theory of emotion. Individuals can experience a mixture of positive affect (PA; e.g., “enthusiastic”) and negative affect (NA; e.g., “afraid”) during a specific period of time (Watson and Tellegen, 1985; Watson and Clark, 1997). The items are scored on a 5-point Likert scale ranging from 1 (*very slightly or not at all*) to 5 (*extremely*). The PANAS has previously demonstrated good internal validity and reliability in athlete populations (Watson et al., 1988) and the Cronbach’s alphas from the current sample were 0.87 for PA and 0.84 for NA in phase 1, and 0.90 for PA and 0.91 for NA in phase 2.

#### Anxiety

The Competitive State Anxiety Inventory-2 (CSAI-2; Martens et al., 1990; Jones and Swain, 1992) was used to assess the intensity and directional interpretation of cognitive and somatic anxiety symptoms. Cognitive anxiety (CA) assesses the mental component of anxiety caused by negative expectations about success or negative self-evaluation (e.g., “I am concerned about losing”) and somatic anxiety (SA) is associated with the physiological or affective component of anxiety (e.g., “My hands are clammy”). The items are scored on a 4-point Likert-scale ranging between 1 (*not at all*) to 4 (*very much so*) for intensity. Further, the directional interpretation of the anxiety symptoms was assessed using a single-item question on a 7-point Likert-scale ranging from −3 (*very negative/debilitative*) to + 3 (*very positive/facilitative*). The CSAI-2 has previously demonstrated good internal validity and reliability in athlete populations (Burton, 1998) and the Cronbach’s alphas from the current sample were 0.88 for CA and 0.89 for SA in phase 1, and 0.88 for CA and 0.89 for SA in phase 2.

### Design

The current study is a cross-sectional, single time-point atemporal design that examines golfers’ approach to competitive situations; an imagined imminent golf competition (phase 1), and an actual future golf competition (phase 2). Specifically, we examine how irrational beliefs interact with cognitive appraisals and challenge and threat to predict affective states (emotions and anxiety) across the two phases. The study was introduced in the form of an online survey to explore the ways in which golfers approach motivated performance situations (golf competition). In phase 1, we adopted an experimental vignette methodology (EVM, Aguinis and Bradley, 2014), where participants were presented with a vignette that represented a real-life scenario in which golfers imagined themselves approaching an imminent golf competition, followed by questionnaires exploring their thoughts and affective states about this event. The vignette was adapted from Skinner and Brewer (2002) to represent a stressful golfing situation, and was presented to players in written form. The personal meaning of the scenario was enhanced by emphasizing the prestige of the tournament, and the composition of the audience. In addition, expectations of other personnel, the final reward, and the presence of other competitors from all across the country emphasized the importance of the event and ensured high levels of pressure. Further, ego-threatening instructions were included, as in line with past golf research (Wilson et al., 2009; Turner et al., 2018) where poor performance represented lack of skill to play at a competitive level. Participants took on average 26 min to complete the survey. The scenario presented to the golfers was as follows:

*You are at an important competition waiting for your name to be announced by the starter at which point you will collect your score card. As you approach the first tee box to start your round, you notice there is a large and dense crowd, more than you have seen before, waiting for you to tee off. This competition is crucial because it is the most prestigious event you have played in and the prize money is the most you’ve competed for. There are high expectations for your performance from friends, family, and the crowd. If you don’t play well then people will think you are not capable of playing at this level and therefore you probably won’t be invited next year. In addition, there is a really strong field of competitors from all over the country. As you step up to the tee, you notice the drastic change in weather conditions*…. *the wind has picked up and it is now raining. You take your position and ready yourself to tee off*…

In phase 2, participants were asked to provide details about their next actual important golf competition and complete questionnaires about their thoughts and affective states in relation to that important event. The aim of phase 2 is to extend phase 1 by examining how golfers’ irrational beliefs interact with cognitive appraisals and challenge and threat to predict affective states in relation to an actual future golf competition. Therefore, the real-life event of an actual upcoming competition allows us to explore the phenomenon in relation to a real-life stressor. This is important, because irrational beliefs are implicated in affectivity different for real vs. imagined stressors (Visla et al., 2016).

### Analytic Strategy

Data for both the phases were examined for missing values. In phase 1, little’s MCAR test revealed that across each variables data between 2.4 and 10.5% were missing at random, *χ*^2^ = 462.55, df = 425, *p* > 0.05. In phase 2, little’s MCAR test revealed that across each variables the data between 2.8 and 4.7% were missing at random, *χ*^2^ = 192.37, df = 169, *p* > 0.05. In the current research, since the missing values were scattered throughout the data, the employment of the deletion technique where missing values are discarded would have resulted in substantial loss of participants, thus reducing the total sample size and further resulting in loss of power (Tabachnick and Fidell, 2007; Baraldi and Enders, 2010). Therefore, we used expectation maximisation (EM) method, a simple and reasonable approach to estimate the missing values (Graham, 2009), and providing a complete data set for the main analyses (Quinton et al., 2018). Further, in line with previous research (e.g., Dixon and Yuen, 1974; Orr et al., 1991; Smith, 2011) the data were checked for outliers and data points with z scores greater than 2 were winsorized which involved replacing extreme values to reduce the influence of outliers on the data. For phase 1, items for DEM (*n* = 15), AWF (*n* = 14), LFT (*n* = 8), DEP (*n* = 13), MR (*n* = 13), MC (*n* = 14), PFC (*n* = 12), EFC (*n* = 14), challenge (*n* = 14), threat (*n* = 7), positive emotions (*n* = 11), negative emotions (*n* = 11), cognitive anxiety (*n* = 9), somatic anxiety (*n* = 10), and directional interpretation (*n* = 11) were windsorized. For phase 2, items for MR (*n* = 10), MC (*n* = 8), PFC (*n* = 8), EFC (*n* = 13), challenge (*n* = 6), threat (*n* = 10), positive emotions (*n* = 8), negative emotions (*n* = 15), cognitive anxiety (*n* = 10), somatic anxiety (*n* = 10), and directional interpretation (*n* = 15) were windsorized.

Prior to the main analyses, since the data was collected from the same participants in regards to the imagined imminent golf competition (phase 1), and an actual future golf competition (phase 2), it was important to examine differences in cognitive appraisals, challenge and threat, affective states (emotions and anxiety) and directional interpretations of anxiety, between the two phases. To compare the means for each dependent variable between the imagined imminent golf competition and the actual future golf competition, three repeated measures multivariate analysis of covariance (MANCOVA) were conducted, one for cognitive appraisals, one for challenge and threat, and one for affective states. In addition, one repeated measure analysis of covariance (ANCOVA) was conducted for directional interpretations of anxiety. Age and handicap were included as covariates in all analyses in both phases, and in phase 2, the number of weeks until the next important competition was also included as a covariate. The result of Shapiro-Wilk for number of weeks, W_(__212__)_ = 0.67, *p* = 0.000, indicated that this variable was not normally distributed. Therefore, the variable was transformed using log transformation to overcome the heteroscedastic errors (i.e., large error variance) associated with the variable and to make it more homogenous (Nevill, 1997).

Main analyses for both phases were conducted in three main stages. First, descriptive statistics and Pearson’s correlations were calculated for all self-report variables to examine associations between cognitive appraisals, irrational beliefs, challenge and threat, and affective states. Second, path analysis was employed in conjunction with bootstrapping procedures to test the hypothesized model using AMOS. Since most of the variables were moderately to strongly correlated, it was possible to introduce a structure to the correlation matrix in accordance with the path diagram (see Figure 1). The model fit was evaluated using the chi-square statistic (*χ*^2^), comparative fit index (CFI), the root-mean-square error of approximation (RMSEA). CFI provides an indication of how the theoretical model better fits the data in comparison to a base model constraining all constructs to be uncorrelated with one another. A non-significant *χ*^2^ and CFI value of 0.90 or above is considered a good fit (Bentler, 1990; Hu and Bentler, 1998; Vandenbergh and Lance, 2000). Further, a RMSEA value of <0.06 indicates a close fit whereas a value < 0.08 is also considered an acceptable fit (Browne and Cudek, 1993). Vandenbergh and Lance (2000) suggest that a cut-off value of 0.10 for RMSEA is acceptable.

**FIGURE 2:**
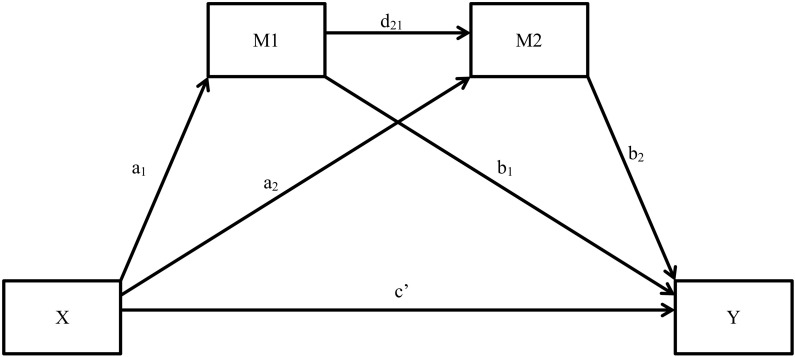
Serial atemporal multiple mediation model with two mediators. X = independent variable; Y = dependent variable; M1, M2 = mediators. a1, a2, b1, b2, d21, c’ = regression coefficients.

Lastly, serial atemporal multiple mediation analysis (SAMM) were conducted using PROCESS version 2.10 for IBM SPSS (Hayes, 2013), to understand the direct and indirect effects of cognitive appraisal, irrational beliefs, and challenge and threat, on affective states. Considering practical implications, PROCESS was employed for multiple mediation as it calculates relevant statistics automatically and efficiently in comparison to structural equation modeling (SEM) programmes such as AMOS that require greater effort and programming skills to gain relevant output. In addition, literature suggests (Hayes et al., 2017) that where the models are entirely based on observed variables, the results yielded from PROCESS and AMOS programmes are substantively identical. Thus, the current methodology is in line with Monteiro et al. (2018), where SEM was used to analyze the relationship between different variables and serial multiple mediation was used to access direct and indirect mediation effects of independent variables on dependent variables. Figure 2 represents a generic model of SAMM with two mediators for illustrative purposes. In the current study in both phases, the independent variable (X) was cognitive appraisals and dependent variables (Y) were affective states (positive or negative emotions, cognitive and somatic anxiety, and directional interpretation of anxiety). Since, there is an established causation from cognitive appraisals to affective states (Lazarus, 1991), in the current research, we treated affective states as the Y variable and cognitive antecedents of affective states as the X and M variables. The data is available on request from the first author of the current study.

## Results

### Repeated Measures Comparison of Phase 1 and Phase 2

#### Cognitive Appraisals

The results of the MANCOVA indicated a significant main effect for cognitive appraisals, Wilks’ Λ = 0.92, *F*(1, 199) = 4.28, *p* < 0.01 η^2^ = 0.08. A significant within-subjects effect was revealed for MC, *F*(1, 199) = 6.02, *p* < 0.01, η^2^ = 0.03, with pairwise comparisons indicating that golfers perceive goals to be less motivationally congruent in anticipation of the imagined imminent golf competition (*M* = 6.94 ± 1.71) compared to an actual future golf competition (*M* = 7.32 ± 1.89). Similarly, a significant within-subjects effect was revealed for PFC, *F*(1, 199) = 9.31, *p* < 0.01, η^2^ = 0.04, with pairwise comparisons indicating that golfers perceived more problem focused coping potential in anticipation of the imagined imminent golf competition (*M* = 7.81 ± 1.85) compared to an actual future golf competition (*M* = 7.59 ± 2.17).

#### Challenge and Threat

The results of the MANCOVA indicated a significant main effect for challenge and threat, Wilks’ Λ = 0.92, *F*(1, 203) = 8.16, *p* < 0.001, η^2^ = 0.07. A significant within-subjects effect was revealed for threat, *F*(1, 203) = 15.68, *p* < 0.01, η^2^ = 0.07, with pairwise comparisons indicating golfers reported greater threat in anticipation of the imagined imminent golf competition (*M* = 2.79 ± 1.10) compared to an actual future golf competition (*M* = 2.22 ± 1.02).

#### Affect

The results of the MANCOVA indicated a significant main effect for emotions, Wilks’ Λ = 0.84, *F*(1, 199) = 8.98, *p* < 0.001, η^2^ = 0.15. A significant within-subjects effect was revealed for negative emotion, *F*(1, 199) = 12.09, *p* < 0.01, η^2^ = 0.06, with pairwise comparisons indicating golfers experienced more negative emotions in anticipation of the imagined imminent golf competition (*M* = 1.87 ± 0.55) compared to an actual future golf competition (*M* = 1.53 ± 0.56). A significant within-subjects effect was revealed for cognitive anxiety, *F*(1, 199) = 8.53, *p* < 0.01, η^2^ = 0.04, with pairwise comparisons indicating golfers reported greater cognitive anxiety in anticipation of the imagined imminent golf competition (*M* = 2.05 ± 0.64) compared to an actual future golf competition (*M* = 1.79 ± 0.58). Also, a significant within-subjects effect was revealed for somatic anxiety, *F*(1, 199) = 34.63, *p* < 0.001, η^2^ = 0.15, with pairwise comparisons indicating golfers experienced more somatic anxiety in anticipation of the imagined imminent golf competition (*M* = 2.04 ± 0.57) in comparison to an actual future golf competition (*M* = 1.60 ± 0.50).

#### Directional Interpretation of Anxiety

The results of the ANCOVA indicated a significant main effect for directional interpretation of anxiety, Wilks’ Λ = 0.91, *F*(1, 199) = 18.51, *p* < 0.01, η^2^ = 0.08, with pairwise comparisons indicating that golfers perceived their anxiety as less facilitative in anticipation of the imagined imminent golf competition (*M* = 1.72 ± 1.26) compared to an actual future golf competition (*M* = 2.01 ± 1.01).

## Phase 1 Results

Table 1 displays descriptive statistics and Pearson’s correlations for all variables.

**TABLE 1:** Mean Scales, Standard Deviations and Correlations among all variables regarding imagined imminent golf competition.

**N = 287**	***M***	***SD***	**Age**	**Handi**	**Exp**	**DEM**	**AWF**	**LFT**	**DEP**	**iBs**	**MR**	**MC**	**PFC**	**EFC**	**Cog App**	**Chall**	**Threat**	**Post Emo**	**Neg Emo**	**CA**	**SA**	**DI**
Age	38.71	15.20	–	0.65^∗∗^	0.53^∗∗^	–0.04	–0.06	–0.18^∗∗^	–0.06	–0.11	–0.11	0.07	–0.01	0.02	0.01	–0.01	−0.14^∗^	–0.06	–0.28^∗∗^	−0.12^∗^	–0.22^∗∗^	0.16^∗∗^
Handi	8.85	7.13		–	0.10	0.04	0.04	–0.11	0.08	0.01	–0.00	–0.07	–0.08	–0.09	–0.10	–0.07	0.05	–0.09	−0.14^∗^	0.06	−0.13^∗^	0.10
Exp	11.86	8.31			–	–0.02	–0.03	–0.08	–0.03	–0.05	–0.09	0.18^∗∗^	0.07	–0.01	0.08	0.04	–0.06	01	−0.12^∗^	–0.08	–0.05	0.05
DEM	20.59	3.82				–	0.76^∗∗^	0.53^∗∗^	0.45^∗∗^	0.81^∗∗^	0.12	–0.19^∗∗^	–0.04	–0.08	–0.10	0.01	0.35^∗∗^	0.08	0.21^∗∗^	0.35^∗∗^	0.19^∗∗^	–0.09
AWF	21.77	4.66					–	0.64^∗∗^	0.54^∗∗^	0.89^∗∗^	0.15^∗^	–0.21^∗∗^	−0.13^∗^	–0.17^∗∗^	–0.17^∗∗^	–0.06	0.46^∗∗^	0.00	0.31^∗∗^	0.42^∗∗^	0.30^∗∗^	–0.16^∗∗^
LFT	23.35	5.48						–	0.46^∗∗^	0.83^∗∗^	0.17^∗∗^	–0.17^∗∗^	–0.11	–0.10	–0.10	0.00	0.30^∗∗^	0.13^∗^	0.22^∗∗^	0.35^∗∗^	0.28^∗∗^	–0.16^∗∗^
DEP	14.85	4.85							–	0.75^∗∗^	0.04	–0.22^∗∗^	–0.11	–0.30^∗∗^	–0.25^∗∗^	–0.22^∗∗^	0.47^∗∗^	–0.21^∗∗^	0.37^∗∗^	0.40^∗∗^	0.25^∗∗^	–0.24^∗∗^
iBs	20.14	3.85								–	0.15^∗^	–0.24^∗∗^	−0.12^∗^	–0.20^∗∗^	–0.19^∗∗^	–0.08	0.48^∗∗^	0.00	0.34^∗∗^	0.46^∗∗^	0.32^∗∗^	–0.20^∗∗^
MR	8.59	1.91									–	0.09	0.15^∗^	0.07	0.44^∗∗^	0.17^∗∗^	0.03	0.32^∗∗^	0.05	0.11	0.10	0.15^∗^
MC	13.78	3.18										–	0.34^∗∗^	0.31^∗∗^	0.77^∗∗^	0.32^∗∗^	–0.33^∗∗^	0.22^∗∗^	–0.28^∗∗^	–0.35^∗∗^	–0.29^∗∗^	0.32^∗∗^
PFC	7.86	1.86											–	0.41^∗∗^	0.69^∗∗^	0.40^∗∗^	–0.33^∗∗^	0.38^∗∗^	–0.28^∗∗^	–0.36^∗∗^	–0.30^∗∗^	0.40^∗∗^
EFC	8.53	2.10												–	0.67^∗∗^	0.38^∗∗^	–0.38^∗∗^	0.28^∗∗^	–0.41^∗∗^	–0.34^∗∗^	–0.37^∗∗^	0.33^∗∗^
Cog App	9.69	1.49													–	0.48^∗∗^	–0.40^∗∗^	0.44^∗∗^	–0.37^∗∗^	–0.38^∗∗^	–0.35^∗∗^	0.46^∗∗^
Chall	4.93	0.70														–	–0.38^∗∗^	0.59^∗∗^	–0.36^∗∗^	–0.33^∗∗^	–0.34^∗∗^	0.49^∗∗^
Threat	2.78	1.09															–	–0.27^∗∗^	0.65^∗∗^	0.72^∗∗^	0.57^∗∗^	–0.45^∗∗^
PostEmo	3.97	0.55																–	–0.24^∗∗^	–0.22^∗∗^	–0.22^∗∗^	0.48^∗∗^
NegEmo	1.86	0.54																	–	0.65^∗∗^	0.71^∗∗^	–0.48^∗∗^
CA	2.05	0.62																		–	0.71^∗∗^	–0.51^∗∗^
SA	2.02	0.55																			–	–0.48^∗∗^
DI	1.66	1.24																				–

*^∗^*p* < 0.05, ^∗∗^*p* < 0.01.*

### Test of the Model

**FIGURE 3:**
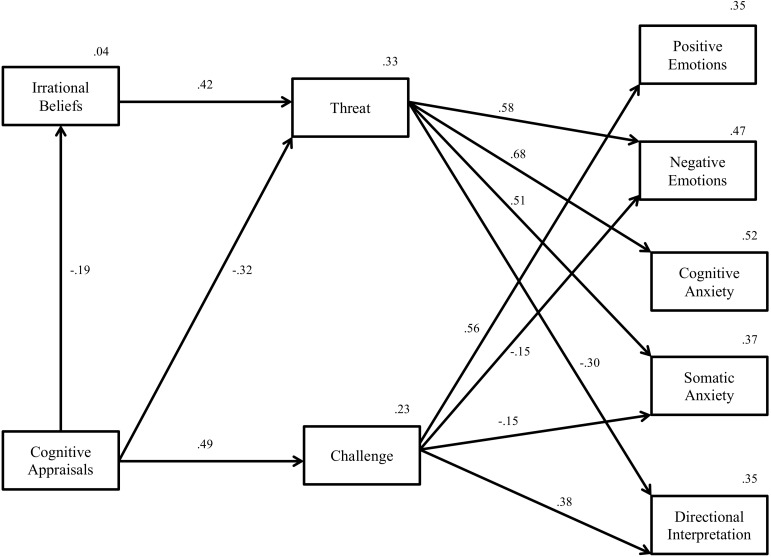
Path analysis testing the theoretical model for imagined imminent golf competition. The model The model indicates all significant paths.

Path analysis revealed that the hypothesized model demonstrated an acceptable fit to the data *χ*^2^(21) = 60.39, *p* < 0.05, CFI = 0.97, RMSEA = 0.08. The standardized path coefficients for each individual path are displayed in Figure 3, demonstrating patterns consistent with study hypotheses. Overall, cognitive appraisals and irrational beliefs accounted for 33% of total variance in threat and 23% of total variance in challenge. With regards to affective states (emotions and anxiety), cognitive appraisals, irrational beliefs, and challenge and threat accounted for 35% of variance in positive emotion, 47% of variance in negative emotion, 52% of variance in cognitive anxiety, 37% of variance in somatic anxiety, and 35% of variance in directional interpretation of anxiety.

### Serial Atemporal Multiple Mediation Analyses (SAMM)

A total of ten SAMM were conducted to assess the direct and indirect effects of cognitive appraisals on affective states (positive and negative emotions, and cognitive and somatic anxiety, and directional interpretation of anxiety), through irrational beliefs, and challenge and threat. Age and handicap were included as covariates. The results of SAMM are presented in Tables 2–4. Total effects for cognitive appraisals on affective states and directional interpretation of anxiety were significant in all the ten mediation models tested. Furthermore, SAMM generated the following results:

**TABLE 2:** Serial atemporal multiple mediation analysis for imagined imminent golf competition.

**Model No.**	**(M1) iBs**	**(M2) appraisals**	**(Y) outcome**	***YR*^2^ = *F*(,) = , *P***	**Total *c* = *t*(df) = , *P***	**Direct c’ = *t* (df) = , *P***	**Indirect# = effect, [to]**	
1		Challenge	PostEmo	*R*^2^ = 0.38 *F*(5, 281) = 35.24, *P* < 0.001	0.16 *t*(283) = 8.12, *P* = 0.00	0.08 *t*(283) = 3.94, *P* = 0.00	Tot = 0.22 [0.15 to 0.30]	Ind1 = −0.01 [−0.03 to 0.002]; Ind2 = 0.24 [0.16 to 0.32]; Ind 3 = −0.001 [−0.01 to 0.008]
2			NegEmo	*R*^2^ = 0.32 *F*(5, 281) = 26.46, *P* < 0.001	−0.13 *t*(283) = −6.85, *P* = 0.00	−0.07 *t*(283) = −3.48, *P* = 0.00	Tot = −0.17 [−0.24 to −0.10]	Ind1 = −0.04 [−0.08 to −0.02]; Ind2 = −0.12 [−0.19 to −0.06]; Ind3 = 0.0003 [−0.005 to 0.01]
3			CogAnxiety	*R*^2^ = 0.35 *F*(5, 281) = 29.77, *P* < 0.001	−0.15 *t*(283) = −6.67, *P* = 0.00	−0.08 *t*(283) = −3.60, *P* = 0.00	Tot = −0.16 [−0.24 to −0.08]	Ind1 = −0.07 [−0.11 to −0.02]; Ind2 = −0.09 [−0.16 to −0.03]; Ind3 = 0.0003 [−0.003 to 0.005]
4			SomAnxiety	*R*^2^ = 0.26 *F*(5, 281) = 20.17, *P* < 0.001	−0.13 *t*(283) = −6.38, *P* = 0.00	−0.07 *t*(283) = −3.29, *P* = 0.00	Tot = −0.15 [−0.24 to −0.08]	Ind1 = −0.04 [−0.08 to −0.01]; Ind2 = −0.11 [−0.18 to −0.05]; Ind3 = 0.0003 [−0.004 to 0.01]
5			DI	*R*^2^ = 0.35 *F*(5, 281) = 30.11, *P* < 0.001	0.38 *t*(283) = 8.87, *P* = 0.00	0.22 *t*(283) = 4.83, *P* = 0.00	Tot = 0.19 [0.12 to 0.27]	Ind1 = 0.02 [0.002 to 0.04]; Ind2 = 0.17 [0.11 to 0.24]; Ind3 = −0.0005 [−0.01 to 0.01]
6		Threat	PostEmo	*R*^2^ = 0.23 *F* (5, 281) = 16.81, *P* < 0.001	0.16 *t*(283) = 8.12, *P* = 0.00	0.14 *t*(283) = 6.72, *P* = 0.00	Tot = 0.05 [0.004 to 0.10]	Ind1 = −0.03 [−0.06 to −0.01]; Ind2 = 0.06 [0.02 to 0.11]; Ind3 = 0.01 [0.003 to 0.03]
7			NegEmo	*R^2^* = 0.48 *F* (5, 281) = 52.02, *P* < 0.001	−0.13 *t*(283) = −6.85, *P* = 0.00	−0.05 *t*(283) = −3.03, *P* = 0.00	Tot = −0.22 [−0.29 to −0.16]	Ind1 = −0.004 [−0.02 to 0.01]; Ind2 = −0.18 [−0.24 to −0.12]; Ind3 = −0.04 [−0.07 to −0.01]
8			CogAnxiety	*R ^2^* = 0.55 *F* (5, 281) = 67.86, *P* < 0.001	−0.15 *t*(283) = −6.67, *P* = 0.00	−0.046 *t*(283) = −2.48, *P* = 0.01	Tot = −0.25 [−0.33 to −0.18]	Ind1 = −0.02 [−0.05 to −0.01]; Ind2 = −0.18 [−0.25 to −0.13]; Ind3 = −0.04 [−0.07 to −0.02]
9			Som Anxiety	*R^2^* = 0.38 *F*(5, 281) = 34.01, *P* < 0.001	−0.13 *t*(283) = −6.38, *P* = 0.00	−0.06 *t*(283) = −2.99, *P* = 0.00	Tot = −0.19 [−0.26 to −0.13]	Ind1 = −0.01 [−0.03 to 0.01]; Ind2 = −0.15 [−0.21 to −0.10]; Ind3 = −0.03 [−0.06 to −0.01]
10			DI	*R*^2^ = 0.32 *F*(5, 281) = 26.47, *P* < 0.001	0.38 *t*(283) = 8.87, *P* = 0.00	0.28 *t*(283) = 6.34, *P* = 0.00	Tot = 0.12 [0.08 to 0.17]	Ind1 = −0.003 [−0.03 to 0.02]; Ind2 = 0.10 [0.06 to 0.15]; Ind3 = 0.02 [0.01 to 0.04]

**TABLE 3:** Regression weights for serial atemporal multiple mediation models for imagined imminent golf competition.

	**Mediators**			**Regression weights**				
		
**Model**	**(M1)**	**(M2)**	**(Y)**	***a*_1_**	***b*_1_**	***d*_21_**	***b*_2_**	***a*_2_**
**No.**	**iBs**		**outcome**					
1		Challenge	PostEmo	–0.46^∗∗^	0.01	0.00	0.39^∗∗^	0.22^∗∗^
2			NegEmo	–0.46^∗∗^	0.03^∗∗^	0.00	–0.19^∗∗^	0.22^∗∗^
3			CogAnxiety	–0.46^∗∗^	0.06^∗∗^	0.00	–0.17^∗∗^	0.22^∗∗^
4			SomAnxiety	–0.46^∗∗^	0.03^∗∗^	0.00	–0.18^∗∗^	0.22^∗∗^
5			DI	–0.46^∗∗^	−0.04^∗^	0.00	0.64^∗∗^	0.22^∗∗^
6		Threat	PostEmo	–0.46	0.02^∗∗^	0.11^∗∗^	–0.11^∗∗^	–0.23^∗∗^
7			NegEmo	–0.46^∗∗^	0.00	0.11^∗∗^	0.28^∗∗^	–0.23^∗∗^
8			CogAnxiety	–0.46^∗∗^	0.02^∗∗^	0.11^∗∗^	0.34^∗∗^	–0.23^∗∗^
9			Som Anxiety	–0.46^∗∗^	0.01	0.11^∗∗^	0.24^∗∗^	–0.23^∗∗^
10			DI	–0.46^∗∗^	0.01	0.11^∗∗^	–0.37^∗∗^	–0.23^∗∗^

**^∗^p* < 0.05, *^∗∗^p* < 0.01.*

**TABLE 4:** Causal chain according to models (X-M-M-Y) for imagined imminent golf competition.

**SAMM**				
Ind1	Cog appraisals	iBs	Post Emo	
**Ind2**	**Cog appraisals**	**Challenge**	**Post Emo**	
Ind3	Cog appraisals	iBs	Challenge	Post Emo
**SAMM**				
**Ind1**	**Cog appraisals**	**iBs**	**Neg Emo**	
**Ind2**	**Cog appraisals**	**Challenge**	**Neg Emo**	
Ind3	Cog appraisals	iBs	Challenge	Neg Emo
**SAMM**				
**Ind1**	**Cog appraisals**	**iBs**	**Cog anxiety**	
**Ind2**	**Cog appraisals**	**Challenge**	**Cog anxiety**	
Ind3	Cog appraisals	iBs	Challenge	Cog anxiety
**SAMM**				
**Ind1**	**Cog appraisals**	**iBs**	**Som anxiety**	
**Ind2**	**Cog appraisals**	**Challenge**	**Som anxiety**	
Ind3	Cog appraisals	iBs	Challenge	Som anxiety
**SAMM**				
**Ind1**	**Cog appraisals**	**iBs**	**DI**	
**Ind2**	**Cog appraisals**	**Challenge**	**DI**	
Ind3	Cog appraisals	iBs	Challenge	DI
**SAMM**				
**Ind1**	**Cog appraisals**	**iBs**	**Post Emo**	
**Ind2**	**Cog appraisals**	**Threat**	**Post Emo**	
**Ind3**	**Cog appraisals**	**iBs**	**Threat**	**Post Emo**
**SAMM**				
Ind1	Cog appraisals	iBs	Neg Emo	
**Ind2**	**Cog appraisals**	**Threat**	**Neg Emo**	
**Ind3**	**Cog appraisals**	**iBs**	**Threat**	**Neg Emo**
**SAMM**				
**Ind1**	**Cog appraisals**	**iBs**	**Cog anxiety**	
**Ind2**	**Cog appraisals**	**Threat**	**Cog anxiety**	
**Ind3**	**Cog appraisals**	**iBs**	**Threat**	**Cog anxiety**
**SAMM**				
Ind1	Cog appraisals	iBs	Som anxiety	
**Ind2**	**Cog appraisals**	**Threat**	**Som anxiety**	
**Ind3**	**Cog appraisals**	**iBs**	**Threat**	**Som anxiety**
**SAMM**				
Ind1	Cog appraisals	iBs	DI	
**Ind2**	**Cog appraisals**	**Threat**	**DI**	
**Ind3**	**Cog appraisals**	**iBs**	**Threat**	**DI**

*Values in bold indicate significant SAMM paths.*

#### Positive Emotion

There were significant indirect effects for cognitive appraisals on positive emotion through challenge (β = 0.24, 95% CI = 0.16–0.31) and through threat (β = 0.06, 95% CI = 0.02–0.11). The indirect effect for cognitive appraisals on positive emotion through irrational beliefs (β = −0.03, 95% CI = −0.06 to −0.01) was significant when threat was included in the model (i.e., model 6). Furthermore, there was a significant indirect effect for cognitive appraisals on positive emotion through both irrational beliefs and threat (β = 0.01, 95% CI = 0.003–0.03). In sum, there was a significant positive direct effect for cognitive appraisals on positive emotion when both mediators (i.e., irrational beliefs and challenge or threat) were included.

#### Negative Emotion

There were significant indirect effects for cognitive appraisals on negative emotion through challenge (β = −0.12, 95% CI = −0.19 to −0.06) and through threat (β = −0.18, 95% CI = −0.23 to −0.12). The indirect effect for cognitive appraisals on negative emotion through irrational beliefs (β = −0.04, 95% CI = −0.08 to −0.02) was significant when challenge was included in the model (i.e., model 2). Furthermore, there was a significant indirect effect for cognitive appraisals on negative emotion through both irrational beliefs and threat (β = −0.04, 95% CI = −0.07 to −0.01). In sum, there was a significant negative direct effect for cognitive appraisals on negative emotion when both mediators (i.e., irrational beliefs and challenge or threat) were included.

#### Cognitive Anxiety

There were significant indirect effects for cognitive appraisals on cognitive anxiety through irrational beliefs when challenge (β = −0.07, 95% CI = −0.11 to −0.02) or threat (β = −0.02, 95% CI = −0.05 to −0.01) were included in the model (i.e., model 3 and 8). The indirect effects for cognitive appraisals on cognitive anxiety were significant through challenge (β = −0.09, 95% CI = −0.16 to −0.04) and also through threat (β = −0.18, 95% CI = −0.25 to −0.13). Furthermore, there was a significant indirect effect for cognitive appraisals on cognitive anxiety through both irrational beliefs and threat (β = −0.04, 95% CI = −0.07 to −0.01). In sum, there was a significant negative direct effect for cognitive appraisals on cognitive anxiety when both mediators (i.e., irrational beliefs and challenge or threat) were included.

#### Somatic Anxiety

There were significant indirect effects for cognitive appraisals on somatic anxiety through challenge (β = −0.11, 95% CI = −0.18 to −0.05) and through threat (β = −0.15, 95% CI = −0.21 to −0.10). The indirect effect for cognitive appraisals on somatic anxiety through irrational beliefs (β = −0.04, 95% CI = −0.08 to −0.01) was significant when challenge was included in the model (i.e., model 4). Furthermore, there was a significant indirect effect for cognitive appraisals on somatic anxiety through both irrational beliefs and threat (β = −0.03, 95% CI = −0.06 to −0.01). In sum, there was a significant negative direct effect for cognitive appraisals on somatic anxiety when both mediators (i.e., irrational beliefs and challenge or threat) were included.

#### Directional Interpretation

There were significant indirect effects for cognitive appraisals on directional interpretation of anxiety through challenge (β = 0.17, 95% CI = 0.11–0.24) and through threat (β = 0.10, 95% CI = 0.06–0.15). The indirect effect for cognitive appraisals on directional interpretation of anxiety through irrational beliefs (β = 0.02, 95% CI = 0.002–0.04) was significant when challenge was included in the model (i.e., model 5). Furthermore, there was a significant indirect effect for cognitive appraisals on directional interpretation of anxiety through both irrational beliefs and threat (β = 0.02, 95% CI = 0.01–04). In sum, there was a significant positive direct effect for cognitive appraisals on directional interpretation of anxiety when both mediators (i.e., irrational beliefs and challenge or threat) were included.

In summary, the data shows that the relationship between cognitive appraisals and affective states is mediated by irrational beliefs and threat in all models, and by irrational beliefs and challenge in some models. In other words, the cognitive appraisals, irrational beliefs and threat are seen as essential antecedents in predicting affective states among golfers.

## Phase 2 Results

Table 5 displays descriptive statistics and Pearson’s correlations for all variables.

**TABLE 5:** Mean Scales, Standard Deviations and Correlations among all variables for actual future golf competition.

***N* = 212**	**M**	**SD**	**Age**	**Handi**	**Exp**	**No. of weeks**	**DEM**	**AWF**	**LFT**	**DEP**	**Total iBs**	**MR**	**MC**	**PFC**	**EFC**	**Cog App**	**Chall**	**Threat**	**Post Emo**	**Neg Emo**	**CA**	**SA**	**DI**
Age	38.55	15.08	–	0.62^∗∗^	0.53^∗∗^	–0.07	–0.04	–0.07	–0.18^∗∗^	–0.07	–0.12	–0.18^∗∗^	–0.04	–0.25^∗∗^	–0.04	−0.16^∗^	–0.10	–0.20^∗∗^	–0.01	–0.27^∗∗^	–0.18^∗∗^	–0.19^∗∗^	0.04
Handi	8.68	7.15		–	0.09	–0.10	0.07	0.10	–0.08	0.14^∗^	0.06	–0.23^∗∗^	–0.18^∗∗^	–0.29^∗∗^	−0.17^∗^	–0.29^∗∗^	–0.12	0.04	–0.08	–0.09	–0.02	–0.12	0.02
Exp	12.81	8.38			–	−0.15^∗^	–0.05	–0.10	–0.13	–0.06	–0.10	−0.16^∗^	0.01	–0.06	–0.04	–0.07	–0.07	–0.08	–0.08	–0.13	–0.10	–0.05	–0.03
No. of weeks	1.16	1.07				–	–0.06	0.00	–0.07	–0.01	–0.04	0.07	–0.02	0.02	0.03	0.02	–0.05	0.14^∗^	0.07	0.12	0.15^∗^	0.15^∗^	–0.04
DEM	20.53	3.83					–	0.77^∗∗^	0.57^∗∗^	0.47^∗∗^	0.82^∗∗^	0.06	−0.14^∗^	–0.07	−0.15^∗^	–0.11	0.13	0.31^∗∗^	0.11	0.10	0.31^∗∗^	0.13	0.00
AWF	21.79	4.75						–	0.67^∗∗^	0.57^∗∗^	0.90^∗∗^	0.04	–0.25^∗∗^	–0.12	−0.16^∗^	–0.18^∗∗^	–0.00	0.43^∗∗^	0.02	0.26^∗∗^	0.40^∗∗^	0.26^∗∗^	−0.15^∗^
LFT	23.59	5.52							–	0.47^∗∗^	0.83^∗∗^	0.14^∗^	–0.22^∗∗^	0.01	–0.11	–0.09	0.14^∗^	0.27^∗∗^	0.12	0.15^∗^	0.29^∗∗^	0.16^∗^	–0.09
DEP	14.90	5.11								–	0.77^∗∗^	0.01	–0.36^∗∗^	–0.13	–0.34^∗∗^	–0.30^∗∗^	−0.15^∗^	0.51^∗∗^	−0.15^∗^	0.39^∗∗^	0.47^∗∗^	0.37^∗∗^	–0.27^∗∗^
Total iBs	20.20	3.98									–	0.08	–0.30^∗∗^	–0.09	–0.23^∗∗^	–0.21^∗∗^	0.03	0.46^∗∗^	0.03	0.28^∗∗^	0.45^∗∗^	0.28^∗∗^	−0.16^∗^
MR	7.82	2.48										–	0.30^∗∗^	0.30^∗∗^	0.21^∗∗^	0.62^∗∗^	0.40^∗∗^	–0.13	0.43^∗∗^	0.03	0.05	0.01	0.20^∗∗^
MC	14.78	3.63											–	0.32^∗∗^	0.47^∗∗^	0.80^∗∗^	0.42^∗∗^	–0.41^∗∗^	0.30^∗∗^	–0.38^∗∗^	–0.38^∗∗^	–0.40^∗∗^	0.44^∗∗^
PFC	7.60	2.19												–	0.53^∗∗^	0.69^∗∗^	0.44^∗∗^	–0.20^∗∗^	0.34^∗∗^	–0.11	−0.14^∗^	−0.17^∗^	0.33^∗∗^
EFC	8.44	2.21													–	0.74^∗∗^	0.41^∗∗^	–0.35^∗∗^	0.33^∗∗^	–0.36^∗∗^	–0.33^∗∗^	–0.40^∗∗^	0.50^∗∗^
Cog App	9.66	1.90														–	0.58^∗∗^	–0.40^∗∗^	0.48^∗∗^	–0.31^∗∗^	–0.30^∗∗^	–0.35^∗∗^	0.51^∗∗^
Chall	4.96	0.73															–	–0.39^∗∗^	0.66^∗∗^	–0.37^∗∗^	–0.25^∗∗^	–0.39^∗∗^	0.54^∗∗^
Threat	2.24	1.00																–	–0.30^∗∗^	0.63^∗∗^	0.73^∗∗^	0.61^∗∗^	–0.40^∗∗^
PostEm	3.98	0.65																	–	–0.27^∗∗^	–0.18^∗∗^	–0.25^∗∗^	0.43^∗∗^
NegEm	1.53	0.53																		–	0.72^∗∗^	0.82^∗∗^	–0.40^∗∗^
CA	1.80	0.57																			–	0.73^∗∗^	–0.40^∗∗^
SA	1.61	0.49																				–	–0.46^∗∗^
DI	1.99	1.00																					–

**^∗^p < 0.05, ^∗∗^p < 0.01.**

### Test of the Model

**FIGURE 4:**
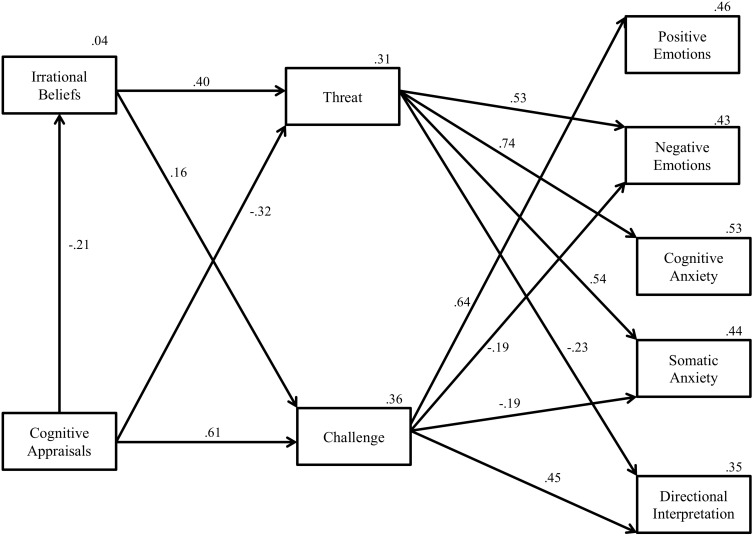
Path analysis testing the theoretical model for an actual future golf competition. The model indicates all significant paths.

Path analysis revealed that the hypothesized model demonstrated an acceptable fit to the data *χ*^2^(31) = 107.31, *p* < 0.05, CFI = 0.94, RMSEA = 0.11. The standardized path coefficients for each individual path are displayed in Figure 4, demonstrating patterns consistent with study hypotheses. Overall, cognitive appraisals and irrational beliefs accounted for 37% of total variance in threat and 57% of total variance in challenge. With regards to affective states (emotions and anxiety), cognitive appraisals, irrational beliefs, and challenge and threat accounted for 46% of variance in positive emotion, 41% of variance in negative emotion, 53% in cognitive anxiety, 40% in somatic anxiety, and 34% in directional interpretation of anxiety.

### Serial Atemporal Multiple Mediation Analysis (SAMM)

A total of ten SAMM analyses were conducted to assess the indirect effects of cognitive appraisals on affective states (positive and negative emotions, cognitive and somatic anxiety, and directional interpretation or anxiety), through irrational beliefs and challenge and threat. Age, handicap and number of weeks to the next important competition were included as covariates. The results of SAMM are presented in Tables 6–8. Total effects of cognitive appraisals on affective states and directional interpretation of anxiety were significant in all the ten mediation models tested. Furthermore, SAMM generated the following results:

**TABLE 6:** Serial atemporal multiple mediation analysis for actual future golf competition.

**Model No.**	**(M1) iBs**	**(M2) appraisals**	**(Y) outcome**	***YR*^2^ = *F*(,) = , *P***	**Total *c* = *t*(df) = , *P***	**Direct c’ = *t* (df) = , *P***	**Indirect# = effect, [to]**	
1		Challenge	PostEmo	*R*^2^ = 0.47 *F*(6, 205) = 30.44, *P* < 0.001	0.17 *t*(207) = 7.89, *P* = 0.00	0.06 *t*(207) = 2.52, *P* = 0.01	Tot = 0.33 [0.24 to 0.43]	Ind1 = −0.01 [−0.04 to 0.01] Ind2 = 0.36 [0.27 to 0.45] Ind3 = −0.02 [−0.04 to −0.002]
2			NegEmo	*R*^2^ = 0.32 *F*(6, 205) = 15.81, *P* < 0.001	−0.10 *t*(207) = −5.55, *P* = 0.00	−0.03 *t*(207) = −1.45, *P* = 0.15	Tot = −0.25 [−0.35 to −0.14]	Ind1 = −0.05 [−0.09 to −0.01] Ind2 = −0.21 [−0.31 to −0.11] Ind3 = 0.01 [0.001 to 0.03]
3			Cog anxiety	*R*^2^ = 0.32 *F*(6, 205) = 16.42, *P* < 0.001	−0.10 *t*(207) = −4.98, *P* = 0.00	−0.040 *t*(207) = −1.76, *P* = 0.08	Tot = −0.20 [−0.31 to −0.09]	Ind1 = −0.08 [−0.15 to −0.03] Ind2 = −0.12 [−0.21 to −0.03] Ind3 = 0.01 [0.0003 to 0.02]
4			Som anxiety	*R*^2^ = 0.31 *F*(6, 205) = 15.63, *P* < 0.001	−0.11 *t*(207) = −6.47, *P* = 0.00	−0.04 *t*(207) = −2.33, *P* = 0.02	Tot = −0.24 [−0.35 to −0.14]	Ind1 = −0.05 [−0.10 to −0.01] Ind2 = −0.20 [−0.30 to −0.10] Ind3 = 0.01 [0.001 to 0.02]
5			DI	*R*^2^ = 0.39 *F*(6, 205) = 22.11, *P* < 0.001	0.30 *t*(207) = 9.34, *P* = 0.00	0.17 *t*(207) = 4.44, *P* = 0.00	Tot = 0.25 [0.15 to 0.35]	Ind1 = 0.02 [0.0005 to 0.06] Ind2 = 0.24 [0.14 to 0.34] Ind3 = −0.01 [−0.03 to −0.001]
6		Threat	PostEmo	*R*^2^ = 0.30 *F*(6, 205) = 14.58, *P* < 0.001	0.17 *t*(207) = 7.89, *P* = 0.00	0.15 *t*(207) = 6.59, *P* = 0.00	Tot = 0.05 [−0.01 to 0.13]	Ind1 = −0.05 [−0.10 to −0.01] Ind2 = 0.08 [0.03 to 0.15] Ind3 = 0.02 [0.003 to 0.04]
7			NegEmo	*R*^2^ = 0.43 *F*(6, 205) = 26.21, *P* < 0.001	−0.10 *t*(207) = −5.55, *P* = 0.00	−0.04 t(207) = −2.18, *P* = 0.03	Tot = −0.22 [−0.31 to −0.14]	Ind1 = 0.003 [−0.02 to 0.03] Ind2 = −0.19 [−0.27 to −0.11] Ind3 = −0.04 [−0.07 to −0.01]
8			Cog anxiety	*R*^2^ = 0.55 *F*(6, 205) = 42.40, *P* < 0.001	−0.10 *t*(207) = −4.98, *P* = 0.00	−0.01 *t*(207) = −0.66, *P* = 0.51	Tot = −0.29 [−0.39 to −0.20]	Ind1 = −0.031 [−0.07 to −0.01] Ind2 = −0.22 [−0.30 to −0.13] Ind3 = −0.05 [−0.08 to −0.02]
9			Som anxiety	*R*^2^ = 0.43 *F*(6, 205) = 25.64, *P* < 0.001	−0.107 *t*(207) = −6.47, *P* = 0.00	−0.049 *t*(207) = −3.18, *P* = 0.00	Tot = −0.22 [−0.31 to −0.15]	Ind1 = −0.002 [−0.03 to 0.02] Ind2 = −0.18 [−0.26 to −0.11] Ind3 = −0.04 [−0.07 to −0.01]
10			DI	*R*^2^ = 0.34 *F*(6, 205) = 17.48, *P* < 0.001	0.30 *t*(207) = 9.34, *P* = 0.00	0.25 *t*(207) = 7.24, *P* = 0.00	Total = 0.09 [0.04 to 0.16]	Ind1 = −0.01 [−0.04 to 0.02] Ind2 = 0.08 [0.03 to 0.14] Ind3 = 0.02 [0.003 to 0.04]

**TABLE 7:** Regression weights for serial atemporal multiple mediation models for actual future golf competition.

	**Mediators**			**Regression weights**				
		
**Model**	**(M1)**	**(M2)**	**(Y)**	***a*_1_**	***b*_1_**	***d*_21_**	***b*_2_**	***a*_2_**
**No.**	**iBs**		**outcome**					
1		Challenge	PostEmo	–0.43^∗∗^	0.01	0.03^∗^	0.51^∗∗^	0.24^∗∗^
2			NegEmo	–0.43^∗∗^	0.03^∗∗^	0.03^∗^	–0.24^∗∗^	0.24^∗∗^
3			Cog anxiety	–0.43^∗∗^	0.06^∗∗^	0.03^∗^	−0.15^∗^	0.24^∗∗^
4			Som anxiety	–0.43^∗∗^	0.03^∗∗^	0.03^∗^	–0.22^∗∗^	0.24^∗∗^
5			DI	–0.43^∗∗^	−.03^∗^	0.03^∗^	0.52^∗∗^	0.24^∗∗^
6		Threat	PostEmo	–0.43^∗∗^	0.04^∗∗^	0.09^∗∗^	–0.16^∗∗^	–0.18^∗∗^
7			NegEmo	–0.43^∗∗^	–0.00	0.09^∗∗^	0.29^∗∗^	–0.18^∗∗^
8			Cog anxiety	–0.43^∗∗^	0.02^∗∗^	0.09^∗∗^	0.36^∗∗^	–0.18^∗∗^
9			Som anxiety	–0.43^∗∗^	0.00	0.09^∗∗^	0.26^∗∗^	–0.18^∗∗^
10			DI	–0.43^∗∗^	0.01	0.09^∗∗^	–0.24^∗∗^	–0.18^∗∗^

*^∗^*p* < 0.05, ^∗∗^*p* < 0.01.*

**TABLE 8:** Causal chain according to models (X-M-M-Y) for actual future golf competition.

**SAMM**				
Ind1	Cog appraisals	iBs	Post Emo	
**Ind2**	**Cog appraisals**	**Challenge**	**Post Emo**	
**Ind3**	**Cog appraisals**	**iBs**	**Challenge**	**Post Emo**
**SAMM**				
**Ind1**	**Cog appraisals**	**iBs**	**Neg Emo**	
**Ind2**	**Cog appraisals**	**Challenge**	**Neg Emo**	
**Ind3**	**Cog appraisals**	**iBs**	**Challenge**	**Neg Emo**
**SAMM**				
**Ind1**	**Cog appraisals**	**iBs**	**Cog anxiety**	
**Ind2**	**Cog appraisals**	**Challenge**	**Cog anxiety**	
**Ind3**	**Cog appraisals**	**iBs**	**Challenge**	**Cog anxiety**
**SAMM**				
**Ind1**	**Cog appraisals**	**iBs**	**Som anxiety**	
**Ind2**	**Cog appraisals**	**Challenge**	**Som anxiety**	
**Ind3**	**Cog appraisals**	**iBs**	**Challenge**	**Som anxiety**
**SAMM**				
**Ind1**	**Cog appraisals**	**iBs**	**DI**	
**Ind2**	**Cog appraisals**	**Challenge**	**DI**	
**Ind3**	**Cog appraisals**	**iBs**	**Challenge**	**DI**
**SAMM**				
**Ind1**	**Cog appraisals**	**iBs**	**Post Emo**	
**Ind2**	**Cog appraisals**	**Threat**	**Post Emo**	
**Ind3**	**Cog appraisals**	**iBs**	**Threat**	**Post Emo**
**SAMM**				
Ind1	Cog appraisals	iBs	Neg Emo	
**Ind2**	**Cog appraisals**	**Threat**	**Neg Emo**	
**Ind3**	**Cog appraisals**	**iBs**	**Threat**	**Neg Emo**
**SAMM**				
**Ind1**	**Cog appraisals**	**iBs**	**Cog anxiety**	
**Ind2**	**Cog appraisals**	**Threat**	**Cog anxiety**	
**Ind3**	**Cog appraisals**	**iBs**	**Threat**	**Cog anxiety**
**SAMM**				
Ind1	Cog appraisals	iBs	Som anxiety	
**Ind2**	**Cog appraisals**	**Threat**	**Som anxiety**	
**Ind3**	**Cog appraisals**	**iBs**	**Threat**	**Som anxiety**
**SAMM**				
Ind1	Cog appraisals	iBs	DI	
**Ind2**	**Cog appraisals**	**Threat**	**DI**	
**Ind3**	**Cog appraisals**	**iBs**	**Threat**	**DI**

*Values in bold indicate significant SAMM paths.*

#### Positive Emotion

There were significant indirect effects for cognitive appraisals on positive emotion through challenge (β = 0.36, 95% CI = 0.27–0.45) and through threat (β = 0.08, 95% CI = 0.03–0.15). The indirect effect for cognitive appraisals on positive emotion through irrational beliefs (β = −0.05, 95% CI = −0.10 to −0.01) was significant when threat was included in the model (i.e., model 6). Furthermore, there was a significant indirect effect for cognitive appraisals on positive emotion through both irrational beliefs and challenge (β = −0.02, 95% CI = −0.04 to −0.002) or threat (β = 0.02, 95% CI = 0.003–0.04). In sum, there was a significant positive direct effect for cognitive appraisals on positive emotion when both mediators (i.e., irrational beliefs and challenge or threat) were included.

#### Negative Emotion

There were significant indirect effects for cognitive appraisals on negative emotion through challenge (β = −0.21, 95% CI = −0.31 to −0.11) and through threat (β = −0.19, 95% CI = −0.27 to −0.11). The indirect effect for cognitive appraisals on negative emotion through irrational beliefs (β = −0.05, 95% CI = −0.09 to −0.01) was significant when challenge was included in the model (i.e., model 2). Furthermore, there was a significant indirect effect for cognitive appraisals on negative emotion through both irrational beliefs and challenge (β = 0.01, 95% CI = 0.001–0.03) or threat (β = −0.04, 95% CI = −0.07 to −0.01). In sum, there was a non-significant negative direct effect for cognitive appraisals on negative emotion when both mediators (i.e., irrational beliefs and challenge) were included.

#### Cognitive Anxiety

There were significant indirect effects for cognitive appraisals on cognitive anxiety through irrational beliefs when challenge (β = −0.08, 95% CI = −0.15 to −0.03) or threat (β = −0.03, 95% CI = −0.07 to −0.01) were included in the model (i.e., model 3 and 8). The indirect effects for cognitive appraisals on cognitive anxiety were significant through challenge (β = −0.12, 95% CI = −0.21 to −0.03) and also through threat (β = −0.22, 95% CI = −0.30 to −0.13). Furthermore, there was a significant indirect effect for cognitive appraisals on cognitive anxiety through both irrational beliefs and challenge (β = 0.01, 95% CI = 0.0003–0.02) or threat (β = −0.05, 95% CI = −0.08 to −0.02). In sum, there was a non-significant negative direct effect for cognitive appraisals on cognitive anxiety when both mediators (i.e., irrational beliefs and challenge or threat) were included.

#### Somatic Anxiety

There were significant indirect effects for cognitive appraisals on somatic anxiety through challenge (β = −0.20, 95% CI = −0.30 to −0.10) and through threat (β = 0.18, 95% CI = −0.26 to −0.11). The indirect effect for cognitive appraisals on somatic anxiety through irrational beliefs (β = −0.05, 95% CI = −0.10 to −0.01) was significant when challenge was included in the model (i.e., model 4). Furthermore, there was a significant indirect path for cognitive appraisals on somatic anxiety through both irrational beliefs and challenge (β = 0.01, 95% CI = 0.001–0.02) or threat (β = −0.04, 95% CI = −0.07 to −0.01). In sum, there was a significant negative direct effect for cognitive appraisals on somatic anxiety when both mediators (i.e., irrational beliefs and challenge or threat) were included.

#### Directional Interpretation

There were significant indirect effects for cognitive appraisals on directional interpretation of anxiety through challenge (β = 0.24, 95% CI = 0.14–0.34) and also through threat (β = 0.08, 95% CI = 0.03–0.14). The indirect effect for cognitive appraisals on directional interpretation of anxiety through irrational beliefs (β = 0.02, 95% CI = 0.0005–0.06) was significant when challenge was included in the model (i.e., model 5). Furthermore, there was a significant indirect path for cognitive appraisals on directional interpretation of anxiety through both irrational beliefs and challenge (β = −0.01, 95% CI = −0.03 to −0.001) or threat (β = 0.02, 95% CI = 0.003–0.04). In sum, there was a significant positive direct effect for cognitive appraisals on directional interpretation of anxiety when both mediators (i.e., irrational beliefs and challenge or threat) were included.

In summary, data analyses demonstrate that the relationships between cognitive appraisals and affective states and directional interpretation of anxiety is mediated by irrational beliefs and challenge and threat in all models. In other words, the interaction of cognitive appraisals, irrational beliefs, and challenge and threat, emerged as antecedent to the golfers’ affective states on approach to both imagined imminent, and actual future golf competitions.

## Discussion

The main aim of the current study was to examine the interaction between cognitive appraisals, irrational beliefs, and challenge and threat, in anteceding pre-competitive affective states (emotions and anxiety) and directional interpretation of anxiety in golfers. To achieve this main aim, two study phases were undertaken where golfers considered an imagined imminent golf competition (phase 1), and an actual future golf competition (phase 2). The current study is the first to investigate how affective states occur through the complex interaction of antecedent cognitive appraisals, irrational beliefs, and challenge and threat, within a specific sporting population.

In accordance with study hypotheses, the results of path analyses across both the study phases revealed that threat was positively associated with negative emotions (H4) and both cognitive and somatic anxiety (H5). Threat was also negatively associated with directional interpretation of anxiety, such that greater threat was associated with less facilitative perceptions of anxiety (H6). In addition, threat was positively associated with irrational beliefs (H2) and negatively associated with cognitive appraisals (H3). Challenge was negatively associated with negative emotions (H4) and somatic anxiety (H5), and positively associated with positive emotions (H4) and more facilitative perceptions of anxiety (H6). Also, cognitive appraisals were negatively associated with irrational beliefs (H1). Further, challenge was positively related to cognitive appraisals (H3), and in phase 2 was positively associated with irrational beliefs (H2), but unrelated to irrational beliefs in phase 1.

In other words, a golfer approaching competition with low cognitive appraisals, that report high irrational beliefs, is more likely to be threatened, and less likely to be challenged. As a result, the golfer will likely experience greater negative emotions and anxiety and is more likely to perceive their anxiety symptoms as less facilitative for their performance in that competition.

The findings of current research support some extant research (e.g., David et al., 2002, 2005) in revealing the interaction between irrational beliefs and cognitive appraisals in the prediction of affective states. The current research extends previous research by investigating and understanding the complex interaction of antecedents to affective states within a golf specific sport setting. David et al. (2002, 2005) did not consider challenge and threat in their studies. Our findings that challenge and threat mediate the relationship between cognitive appraisals and affective states alongside irrational beliefs is an important extension of our knowledge of how affective states occur. Also, our research takes into account the interpretation of anxiety, previously unexplored in research. The current research also makes methodological advancements by using more sophisticated analytical procedures (SEM and SAMM).

The inclusion of challenge and threat in the current study, alongside irrational beliefs and cognitive appraisals, is a particularly important extension of past research because it more comprehensively reflects the antecedents of affective states in anticipation of personally relevant situations. Researchers have found irrational beliefs to be positively associated with threat (Dixon et al., 2016; Evans et al., 2018), but the current study develops this research by offering an integration of cognitive appraisals, irrational beliefs, and challenge and threat. The finding that challenge and threat are associated differentially with affective states is in line with the postulations of prominent theories (e.g., Skinner and Brewer, 2004; Jones et al., 2009). That is, challenge was associated with positive affective states, and more facilitative perceptions of anxiety, whilst threat was related to negative affective states, and less facilitative perceptions of anxiety. The findings concerning anxiety in the current study are in line with previous research that demonstrates threat to be associated with greater cognitive and somatic anxiety and a more debilitative interpretation of anxiety responses compared to a challenge (e.g., Williams et al., 2010; Quested et al., 2011; Moore et al., 2012). Specifically, Moore et al. (2012) found that the golfers who received challenge instructions reported lower levels of cognitive anxiety compared to golfers who received threat instructions. In addition, golfers who received challenge instructions interpreted anxiety to be more facilitative for their performance in comparison to golfers who received threat instructions.

Beyond the bivariate associations emerging from path analyses, SAMM provided some important evidence concerning the mechanisms that could explain the relationships between cognitive appraisals and affective states. There were significant indirect effects across both study phases, implying that the association between cognitive appraisals and affective states was mediated by irrational beliefs and threat (H7). This is in support of previous research in which irrational beliefs are associated with cognitive appraisals (David et al., 2002, 2005), and where higher irrational beliefs are associated with greater threat, and lesser challenge (Dixon et al., 2016). That irrational beliefs and threat mediated the relationship between cognitive appraisal and affective states in serial suggests that it is the interaction between cognitive appraisals, irrational beliefs, and threat, that is particularly important for understanding anticipatory affective states on approach to competitive golf situations.

With regards to challenge, there were some differences between phase 1 and phase 2 in the serial multiple atemporal mediation results. With challenge in the mediation model, at phase 1 no significant serial mediation was found for any affective outcomes, although simple mediation was revealed. However, in phase 2, significant serial mediation was found for all affective states, showing that irrational beliefs and challenge (H7) in serial mediated the association between cognitive appraisals and affective states. This lack of serial mediation in phase 1 could be due to a variety of factors. First, there is no significant relationship between irrational beliefs and challenge in phase 1, revealed in bivariate correlations (Table 1), and in the path analysis. Second, in phase 1 participants approached an imagined competition scenario, whereas in phase 2 they approached a real future competition. It might be that the imagined event induced greater psychological pressure than what the participants might face in their next actual competition. In phase 1, we induced pressure using ego-threat, but in phase 2, we did not induce pressure at all. Therefore, challenge might have been more salient in phase 2 where a participant’s next competition might be one in which they are facing less pressure to perform because some participants are unlikely to be performing under the pressured conditions reflected in phase 1. Therefore, challenge is more likely to emerge on approach to a less pressured competition (Blascovich and Mendes, 2000).

The finding that mediation and bivariate associations differed across phase 1 and phase 2 is also echoed in the phase 1-phase 2 tests of differences, and differences in the strength of relationships between the two phases, reported in the results. Specifically, the results revealed that golfers appraised the imagined imminent competition as less motivationally congruent and perceived greater problem focused coping potential during phase 1 than in phase 2. Also, the golfers reported greater threat greater negative emotions and anxiety in phase 1. PFC reflects the potential to act directly on the situation with the purpose of changing the situation or bringing it in accordance with one’s desires (Lazarus, 1999). However, in the current study, PFC is unrealistic for the golfers because the imagined competition is imminent and unchangeable. For instance, if a golfer perceives the situation to be incongruent with his or her goals, focuses on problem focused coping and evaluates the competition as a threat, then he or she is more likely to experience greater negative emotions and anxiety before an imminent golf competition.

The phase 1-phase 2 differences were unexpected and contrary to our hypotheses (H8, 9). We expected golfers to experience stronger negative emotions during phase 2 (H9), and we expected stronger associations between variables in phase 2 (H8), because research indicates that real events are more stressful and should elicit bivariate associations (Visla et al., 2016). It is important however to consider past literature, which suggests that temporal proximity is an important factor when measuring responses to stressful events. For instance, research has extensively investigated the temporal patterning of competitive anxiety (Cerin et al., 2000) and the findings of the studies revealed that the intensity of the somatic component of competitive anxiety increases as competition nears (Slaughter et al., 1994), whereas the cognitive anxiety component can increase (Swain and Jones, 1992; Slaughter et al., 1994) or remain stable (Caruso et al., 1990) on approach to competition. Our findings that affective states were lower in the real event (phase 2) in comparison to the imminent imagined situation (phase 1) could be because the next event for each participant varied in proximity ranging from a few days to months.

The results of the present study indicate the importance of using various procedural and data analytical methods to investigate the associations between cognitive appraisals, irrational beliefs, challenge and threat, and affective states. Although, there were some differences between phases 1 and phase 2, overall path analytical and atemporal mediational models were broadly consistent across both the phases. The findings of the current paper may have some important theoretical implications, in part because we offer a more complex model than has previously need proposed and tested (e.g., David et al., 2002). It is essential and advantageous to consider cognitive appraisals, irrational beliefs, and challenge and threat, in the occurrence of affective states. The model proposed and tested in the current study provides a more accurate and comprehensive explanation concerning the antecedents of affective states on approach to competitive situations. Importantly, cognitive appraisals and irrational beliefs are seen as co-occurring simultaneously rather than occurring in a sequential and fixed order (Ziegler, 2001).

The consistency in SEM and SAMM results between phases 1 and 2 demonstrate the utility of experimental vignettes that represent real-life golf scenarios. The current research has not investigated REBT interventions *per se*, however, it has provided useful information for the readers with regards to potential practical implications. That is, by having golfers imagine approaching an upcoming competition, we were able to identify their beliefs and trigger affective states similar to what was reported for a real golf competition. Thus, practitioners in the field can encourage athletes to imagine upcoming situations in order to trigger cognitive appraisals and irrational beliefs for the purposes of more accurate assessment and intervention. Indeed, in REBT Rational Emotive Imagery (REI) is an oft-used technique (Maultsby, 1971) with athletes (Turner and Bennett, 2018). REI involves athletes visualizing the situation that elicits unhealthy negative emotions and then emotional change is brought about by encouraging them to change their irrational beliefs into rational beliefs.

Researchers have not yet investigated the effects of REI within sporting performance, but motivational general arousal (MG-A) imagery has been suggested as an effective intervention for the enhancement of athletes overall affect experiences and interpretation of pre-competitive symptoms (Mellalieu et al., 2009). Clearly, there is some overlap between MG-A imagery and REI, where imagery focuses upon the emotional experiences associated with stress, anxiety and arousal (Vadocz et al., 1997). However, in MG-A imagery the athletes are asked to imagine arousal reducing images (e.g., imagine oneself in a relaxed place) whereas, in REI the athletes are asked to alter their irrational beliefs in order to change their unhealthy emotional responses to the imagined situation. Further, imagery has been used in research to manipulate challenge and threat (Hale and Whitehouse, 1998; Williams et al., 2010; Williams and Cumming, 2012) and deemed as a useful strategy to help athletes evaluate the competitive situation as a challenge prior to their performance. Additionally, the findings of the current research have established associations between cognitive appraisals, irrational beliefs, and challenge and threat (David et al., 2002; Dixon et al., 2016; Evans et al., 2018). Therefore, practitioners can promote the use of REI combined with MG-A imagery with athletes during consultation. For instance, athletes can be asked to imagine themselves in events or situations (A) that obstruct their goals (G), and trigger unhealthy emotional and behavioral consequences (C), depending on their beliefs about the self, others, and the world in relation to the situation (A). If the athletes beliefs (B) are irrational (rigid, illogical, and extreme) then the practitioner can help them change their irrational beliefs into rational beliefs (flexible, logical, and non-extreme), which in turn can influence athletes to appraise the competition as a challenge, thus, leading to healthy emotional and behavioral responses (C) prior competition. Thus, similar to the imaged situations, REI can be a useful practical tool for practitioners to use with athletes to encourage healthy affective states among athletes in competitions (Ellis and Dryden, 1997).

The current research is not without its limitations. The primary limitation is that we adopted a cross-sectional single time point atemporal design. Cognitions and affective states change in the lead up to important events (e.g., Skinner and Brewer, 2002), and cognitive appraisals are most accurately considered to be iterative, rather than static and singular occurrences (Blascovich and Mendes, 2000; Lerner and Keltner, 2000; Schneider, 2008). Therefore, future research should explore the role that irrational beliefs play in the temporal changes in cognitive appraisals and affective states in the lead up to a sport competition.

Furthermore, the current research uses self-report measures, which can result in biases when investigating cognitive appraisals (e.g., Paunonen and LeBel, 2012). It is possible that the hypothetical scenario in the current paper influenced appraisals unconsciously, outside of the conscious awareness of the participants. Indeed, it may be that only some aspects of cognitive appraisal are consciously accessible with an even smaller section of those perceptions considered acceptable to report by individuals (e.g., Greenwald and Banaji, 1995; LeDoux, 1998; Blascovich and Mendes, 2000; Quigley et al., 2002). To overcome such a limitation, future research using longitudinal designs could investigate emotional experience using more objective psychophysiological markers (see Jones et al., 2009). Also, future researchers would benefit from the development of a sport specific measure for primary and secondary cognitive appraisals. Further, the current research lacks objective measure of sport performance, and researchers should aim to explore how irrational beliefs and cognitive appraisals interact to predict affective states, and in turn, athletic performance. The current sample also involves higher proportion of male golfers in comparison to females. The sex-imbalance with the sport of golf, with 15% of golf club members being female (England Golf, 2018) makes it difficult to make comparisons, therefore future research should look at recruiting equal numbers of males and females for a detailed comparisons (Turner et al., 2018b). In addition, the substantial time delay from completion of the questionnaires to the next competition for some golfers meant that there is great variability in the time to event data. Indeed, due to the variability, we transformed the variable number of weeks to allow us to include it in the analyses (make it more homogenous). Nevertheless, future research might consider recruiting participants within the same time proximity to the next competition to understand the phenomenon in a more homogenous data set. Lastly, within current research, the participants were not recruited based upon a specific range of handicap. The current research aimed to recruit golfers competing at all different levels being club, amateur golfers and professional golfers. Hence, the participants differed across a wide range of handicaps. However, the main aim of the current research was to make an initial investigation concerning how cognitive appraisals, irrational beliefs, and challenge and threat, relate to affective states among competitive golfers. Future researcher could restrict the handicap to more elite athletes and examine the differences between low and high handicap golfers.

## Conclusion

In conclusion, the findings of the current study indicate that irrational beliefs interact with cognitive appraisals and challenge and threat to determine affective states within golfers. The data shows that the relationship between cognitive appraisals and affective states is mediated by irrational beliefs and challenge or threat. In other words, cognitive appraisals, irrational beliefs, and challenge and threat, are seen as interacting antecedents to pre-competitive affective states among golfers. It is hoped that this study stimulates further research and discussion concerning cognitive appraisal in anticipation of competitive situations.

## Data Availability Statement

The datasets generated for this study are available on request to the corresponding author.

## Ethics Statement

This study was carried out in accordance with the recommendations of “Staffordshire University Ethics Committee” with written informed consent from all subjects. All subjects gave written informed consent in accordance with the Declaration of Helsinki. The protocol was approved by the “Staffordshire University Ethics Committee.”

## Author Contributions

NC, MT, and MS conceived the research idea, and structured and drafted the manuscript. NC collected and analyzed the data. MT and MS edited the manuscript and made comments on the final version.

## Conflict of Interest

The authors declare that the research was conducted in the absence of any commercial or financial relationships that could be construed as a potential conflict of interest.
